# The Role of Oxidative Stress in Physiopathology and Pharmacological Treatment with Pro- and Antioxidant Properties in Chronic Diseases

**DOI:** 10.1155/2020/2082145

**Published:** 2020-07-23

**Authors:** Andrés García-Sánchez, Alejandra Guillermina Miranda-Díaz, Ernesto Germán Cardona-Muñoz

**Affiliations:** Department of Physiology, University Health Sciences Center, University of Guadalajara, Guadalajara, Jalisco, Mexico

## Abstract

Oxidative stress (OS) has the ability to damage different molecules and cellular structures, altering the correct function of organs and systems. OS accumulates in the body by endogenous and exogenous mechanisms. Increasing evidence points to the involvement of OS in the physiopathology of various chronic diseases that require prolonged periods of pharmacological treatment. Long-term treatments may contribute to changes in systemic OS. In this review, we discuss the involvement of OS in the pathological mechanisms of some chronic diseases, the pro- or antioxidant effects of their pharmacological treatments, and possible adjuvant antioxidant alternatives. Diseases such as high blood pressure, arteriosclerosis, and diabetes mellitus contribute to the increased risk of cardiovascular disease. Antihypertensive, lipid-lowering, and hypoglycemic treatments help reduce the risk with an additional antioxidant benefit. Treatment with methotrexate in autoimmune systemic inflammatory diseases, such as rheumatoid arthritis, has a dual role in stimulating the production of OS and producing mitochondrial dysfunction. However, it can also help indirectly decrease the systemic OS induced by inflammation. Medicaments used to treat neurodegenerative diseases tend to decrease the mechanisms related to the production of reactive oxygen species (ROS) and balance OS. On the other hand, immunosuppressive treatments used in cancer or human immunodeficiency virus infection increase the production of ROS, causing significant oxidative damage in different organs and systems without widely documented exogenous antioxidant administration alternatives.

## 1. Introduction

Oxidative stress (OS) is characterized by the imbalance between the production and degradation of reactive oxygen species (ROS) or reactive nitrogen species (RNS) [1]. ROS are molecules whose chemical makeup gives them high reactivity and can come from the metabolism of oxygen or nitrogen. ROS and RNS can be free radicals such as the superoxide radical (O_2_^·-^), hydroxyl radical (OH^·^), and nitric oxide (NO^·^). However, other nonfree radicals can also be found, such as hydrogen peroxide (H_2_O_2_) and peroxynitrite (ONOO^−^) [2]. ROS produce enzymatic reactions within the mitochondria characterized by the reduction of oxygen through the electron transport chain [3]. In addition, the endoplasmic reticulum and peroxisomes are other sources of ROS [4, 5]. Different cellular processes such as protein phosphorylation, activation of transcription factors, immunity, and apoptosis depend on the cellular concentration of ROS [6].

The main endogenous antioxidant enzymes that neutralize ROS are superoxide dismutase (SOD), catalase (Cat), and glutathione peroxidase (GPx) [7]. SOD belongs to a group of metalloenzymes that transforms O_2_^·-^ into oxygen and H_2_O_2_ [8]. Three forms of SOD are known in mammals: cytoplasmic SOD (SOD1), mitochondrial SOD (SOD2), and extracellular SOD (SOD3) [9]. ROS can be neutralized by other nonenzymatic molecules with free radical scavenging properties such as vitamins, melatonin, and glutathione (GSH) [10]. When antioxidant defenses fail to properly neutralize ROS, ROS remain in the body longer and oxidize susceptible biomolecules [11]. Excessive levels of ROS can damage cellular proteins, membrane lipids, and nucleic acids, causing damage to proper cellular function [11]. The NO^·^ radical is an endothelium-dependent mediator in vascular vasorelaxation. NO^·^ is produced normally by the enzyme nitric oxide synthase (NOS) [12]. In OS conditions, NO^·^ reacts with the radical O_2_^·-^ to generate ONOO^−^ causing endothelial damage [13].

**Figure 1:**
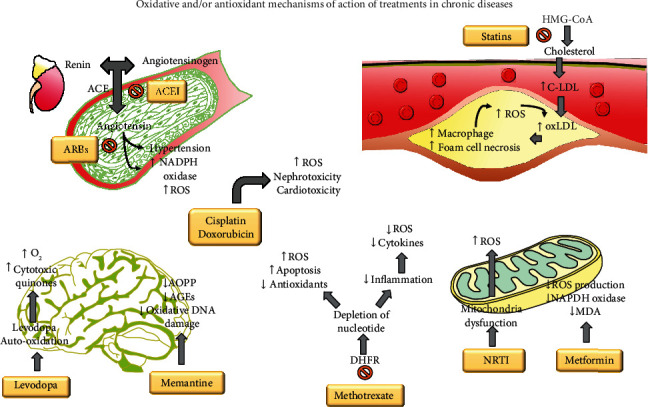
Oxidative and/or antioxidant mechanisms of action of treatments in chronic diseases. Description of how different drug mechanisms affect the oxidative status. Antihypertensive and statin treatment decrease oxidative stress by restoring the endothelial function. Antineoplastic (cisplatin, doxorubicin) and nucleoside or nucleotide reverse transcriptase inhibitor (NRTI or NtRTI) treatment causes the most oxidative damage in patients in the long term. Methotrexate can cause increased OS and apoptosis; at the same time, inflammation-mediated OS production decreases. Levodopa metabolism may increase cytotoxicity in the brain. Metformin and memantine may decrease the oxidative stress.

The lipoperoxidation (LPO) process is a mechanism of damage produced by OS on lipids. LPO is characterized by having carbon-carbon double bonds, especially polyunsaturated fatty acids. The main LPO products are hydroperoxides, such as propanal, hexanal, 4-hydroxynonenal, and malondialdehyde (MDA) [14]. Other LPOs are isoprostanes from nonenzymatic oxidation of essential fatty acids, such as arachidonic acid [15]. Additionally, ROS can damage the DNA structure when they react with guanine bases. Guanine oxidation commonly forms 8-hydroxy-2′-deoxyguanosine (8-OHdG) or 8-oxo-7,8-dihydro-2′-deoxyguanosine (8-oxodG) [16]. These metabolites under normal conditions are repaired by the enzyme oxoguanine glycosylase (hOGG1) and are known jointly, like biomarkers of the OS [17]. OS is present in various chronic diseases, which can contribute to its progression [18]. OS and the inflammatory process are closely linked to each other and contribute to the tissue damage of some autoimmune diseases such as rheumatoid arthritis [19]. OS is linked to hyperglycemia and the progression of type 2 diabetes mellitus (DM) [20]. The participation of OS in cardiovascular disease is mainly attributed to its effects on hypertension and the formation of atheroma leaflets [21, 22]. The pathological development of other chronic diseases such as neurodegenerative diseases [23], cancer [24], or infection by the human immunodeficiency virus (HIV) is related to increased production of ROS [25]. On the other hand, exogenous factors, such as the recommended pharmacological treatments for certain chronic pathologies, have the ability to alter the production of ROS [2]. The purpose of this small review is to describe the role that OS plays in different pathological processes (atherosclerosis, high blood pressure, DM, rheumatoid arthritis, cancer, HIV, and some neurodegenerative diseases). The prooxidant or antioxidant effects of some pharmacological management alternatives will be briefly described (Figure 1).

### 1.1. Oxidative Stress in Atherosclerosis

Atherosclerosis is a chronic disease characterized by inflammation, the manifestation of which occurs in the vascular system. Atherosclerosis is the main origin of cardiovascular disease (CVD) in developed countries of the world [26]. Atherosclerosis represents the development of vascular lesions or plaque deposition in the blood vessels after the response of endothelial damage produced by the inflammation/oxidation processes [27]. Plaque is mainly made up of blood cells, foam cells, lipids, and proteins accompanied by calcium accumulation, favoring vascular expansion, vascular blockage, and inhibition of vascular blood flow, which leads to the explosion of the vascular wall [28, 29]. In CVD, blockage and rupture of the atherosclerotic coronary arteries cause myocardial infarction, while blockage of the carotid arteries causes stroke [30]. Endothelial damage is related to risk factors for the heart and blood vessels such as DM, high blood pressure, nicotine use, lipid disorder, obesity, and metabolic disorders. Impaired endothelial physiological functions are observed during the early stages of atherosclerotic lesions due to oxidative damage [31]. The renin-angiotensin system (RAS) plays an essential role in the advancement of atherosclerosis by influencing endothelial physiology, inflammatory reactions, thrombosis, and oxidative lesions [32]. Angiotensin II (Ang II) causes oxidative damage in the vascular system by inducing the generation of ROS by activating NADPH oxidase with the ability to oxidize cellular biomolecules, including lipids, lipoproteins, and DNA, leading to endothelial deterioration [33].

### 1.2. Management for Atherosclerosis and Oxidative Stress

Hypercholesterolemia is considered the main trigger for atherosclerosis. Therefore, the control of lipoprotein levels through the administration of statins is one of the main management alternatives to reduce the risk of atherosclerosis [34]. Statins antagonize the activity of the enzyme hydroxy-methylglutaryl-coenzyme A (HMG-CoA) reductase, decreasing the production of intracellular cholesterol and decrease of liver LDL receptors [35]. Statins show pleiotropic effects on endothelial function, inhibition of thrombus gene activity, the stability of atherosclerosis plaques, and decreased inflammation and OS [36]. Statins have been shown to have antioxidant effects on redox signaling of vascular and myocardial tissue by modifying NADPH oxidase activity [37]. Statins show effects on eNOS and decreased LPO [38]. Treatment of patients with simvastatin has protective effects on lipoprotein oxidation [39]. However, the metabolism of statins generates ROS and produces toxicity in various tissues, including skeletal muscle and liver damage [40, 41]. The activity of simvastatin and lovastatin inhibits the complete II, III, IV, and V of the electron transport chain, whereas fluvastatin and cerivastatin only inhibit the V complex, thus causing mitochondrial dysfunction [42]. Eight weeks of simvastatin management is sufficient to cause mitochondrial respiration dysfunction in muscle [43].

### 1.3. Adjuvant Antioxidants in Atherosclerosis

Different antioxidant compounds have been used as adjuvant therapy in chronic diseases (Table 1). The antioxidant N-acetylcysteine has been reported to suppress accelerated atherosclerotic events in mouse models with inactivated ApoE [44]. The vitamin D analog (paricalcitol) was also reported to improve oxidative vascular injury by suppressing the activity of ROS-generating enzyme NADPH oxidase, inflammatory mediators, and regulating the antioxidant defense system in ApoE-deficient mice [45]. On the other hand, polyphenols are common antioxidant nutrients, mainly derived from fruits, vegetables, tea, coffee, cocoa, mushrooms, drinks, and traditional medicinal herbs [46, 47]. The classification of polyphenols mainly includes flavonoids (60%), phenolic acids (30%), and other polyphenols, including stilbenes (resveratrol) and ligands, attached to at least one aromatic ring in one or more HO^·^ functional groups [46]. Flavonoids are the most studied group of polyphenols; they are divided into six subclasses: flavonols, flavones, flavanones, flavanols, anthocyanins, and isoflavones. Phenolic acids are divided into two subclasses, benzoic acid and cinnamic acid. Stilbenes in plants act as antifungal phytoalexins and are rare in the human diet [47].

### 1.4. Oxidative Stress in Hypertension

High blood pressure is the most common cardiovascular risk factor and contributor to global morbidity and mortality [48]. High blood pressure is a complex condition. Approximately 90% of cases are classified as essential hypertension, where the precise cause is unknown [49]. Hypertensive stimuli, including salt, hyperactivity of the RAS system, OS, and inflammation lead to the initial elevation of blood pressure, mainly due to central actions and also due to endogenous hormones such as Ang II and aldosterone, resulting in protein modification. The altered proteins are no longer recognized as their own (they serve as neoantigens), and the T cells are activated. T cell derived signals promote macrophage (and other inflammatory cells) entry into the vasculature and kidney, resulting in the release of inflammatory cytokines. In the vasculature, activated T cells promote vasoconstriction and remodeling, along with promoting sodium and water retention in the kidney, causing more severe hypertension [50]. Chronic inflammation has the ability to trigger OS that is associated with high blood pressure. Against the background of Ang II-induced hypertension, T cells express high levels of p47phox, p22phox, and NOX2, components of NOX2 oxidase.

Furthermore, adoptive transfer of NADPH oxidase-deficient T cells results in decreased O_2_^·-^ production and arterial hypertension in response to Ang II [51]. Ang II is one of the main vasoactive signaling molecules involved in ROS generation and participates in increased expression and activity of one of the main ROS generators, NADPH oxidase [52, 53]. The highest production of Ang II occurs in hypertensive conditions [54]. In addition, to intrarenal vasoconstriction, high levels of Ang II have deleterious effects on necrotic and apoptotic changes in kidney tissue during the reperfusion period. Ang II downregulates the SR-BI HDL receptor in proximal tubular cells [55]. Statins were developed to inhibit cholesterol synthesis by blocking HMG-CoA reductase. However, within their pleiotropic effects, these drugs are anti-inflammatory and can produce a small reduction in systolic blood pressure in hypercholesterol patients. The effect is greater on patients with higher blood pressure [56].

### 1.5. Oxidative Stress in Antihypertensive Treatment

First-line management to treat high blood pressure includes angiotensin-converting enzyme inhibitors (ACEI), angiotensin receptor blockers (ARB), calcium channel blockers (CCB), and beta-blockers (BB) [57]. The control of hypertension is associated with the regulation of Ang II activation, which contributes to decreased OS independently of antihypertensive therapy [58]. Antihypertensive treatment with ACEI has been shown to have antioxidant effects. Studies on the effects of enalapril on OS in the kidney and heart of rats with hypertension show that enalapril increases total antioxidant activity and decreases LPO levels in both organs [59, 60]. Other experimental studies show that captopril decreases H_2_O_2_ and MDA levels in hyperglycemic conditions [61]. Telmisartan effectively controls blood pressure and improves fibrosis and vascular remodeling. Additionally, telmisartan exerts protective vessel effects by inhibiting the TGF-*β*1/Smad3 pathway associated with antihypertensive and antioxidant effects [62].

The antioxidant effects of ARB and BB are very similar to those of ACEI; olmesartan attenuates the concentration of TBARS and H_2_O_2_ in obese mice [63]. Eight-week treatment with candesartan or valsartan reduces urinary 8-isoprostanes and 8-OHdG levels compared to treatment with trichlormethiazide [64]. Valsartan treatment also decreases nitrosative stress in patients with type 2 DM [65]. Medium-term treatment with atenolol combined with thiazide hydrochloride decreases MDA levels and increases the concentration of SOD, GSH, and vitamins E and C [66]. Long-term treatment with metoprolol or carvedilol has been shown to decrease LPO levels in patients with heart failure [67]. The reduction of BB use in OS is not limited to plasma or serum. Studies show that carvedilol can also decrease myocardial LPO levels in patients with dilated cardiomyopathy [68].

The CCB are an important antihypertensive group. The dihydropyridine ring through which they can be considered as weak antioxidants is due to their ability to react with peroxyl radicals [69]. Amlodipine shows the ability to reduce isoprostane concentration in patients with type 2 DM [70]. Other BCC, such as nifedipine and lacidipine, have been shown to be protective in the formation of LDL-oxidized lipoprotein [71].

### 1.6. Adjuvant Antioxidants in Arterial Hypertension

Diet is the main source of exogenous antioxidants. Among exogenous antioxidants, polyphenols, vitamins (C and E and *β*-carotene), and minerals stand out. Components like Se, Zn, Fe, Mn, and Cu favor the organism in the elimination of excessive free radicals through adequate enzymatic proteins [72]. Polyphenols can block Ang II-stimulated positive regulation of various NADPH oxidase (NOX) subunits, including NOX1 and p22phox (an essential component of NOX) and associated OS [73]. Some research reveals that systolic blood pressure in hypertensive patients improves after eating foods rich in polyphenols [74]. The combination of dietary flavonoids and antihypertensive drug therapy based on telmisartan or captopril can improve blood pressure, lipid profile, obesity, and inflammation in young hypertensive patients [75].

### 1.7. Oxidative Stress in Diabetes Mellitus

DM is known as an OS disorder caused by the imbalance between the formation of free radicals and the capacity of the body's natural antioxidants. Glucose fluctuations are essential in the pathogenesis of DM. OS plays an important role in the complications of developing DM [76]. OS is directly influenced by fluctuations in glucose. Postprandial glucose fluctuations or any type of glucose oscillation cause greater OS than chronic hyperglycemia. The length and severity of chronic hyperglycemia and regularly occurring acute glucose changes are the main components of glycemic disorders [77]. Hyperglycemia induces ROS production. In type 2 DM, when the *β* cells are still intact and functional, the presence of ROS produces OS in the *β* cells, which leads to lower levels of insulin secretion [77]. The radical O_2_^·-^ is a type of ROS of particular interest in DM, because it has been shown to be elevated in *in vitro* and *in vivo* studies [77]. There are many sources of OS in DM including enzymatic, nonenzymatic, and mitochondrial pathways. The OS increase in DM occurs due to multiple factors [78]. The most dominant oxidizing factor is the autooxidation of glucose, which results in the development of free radicals. Other factors are unbalanced cellular reduction/oxidation and reduced antioxidant defenses (reduced levels of cellular antioxidants and reduced enzyme activity against free radicals) [79]. Due to the high levels of glucose in DM, the generation of O_2_^·-^ triggers multiple pathways, with the greater formation of polyols, higher flow of the hexosamine pathway, and activation of the protein kinase C isoform [80]. Mitochondria are integrative critiques of energy production, ROS generation, signaling transduction, and apoptosis in DM. Within the mitochondrial dynamics highlights the importance of the fusion and fission processes in mitochondrial homeostasis [81]. Mitochondrial fusion appears to be beneficial because it distributes metabolites, proteins, and DNA through the mitochondrial network. Excessive mitochondrial fission can be harmful because it causes fragmented mitochondria to accumulate with an impaired electron transport chain with the ability to increase mitochondrial ROS in cells [82]. In 2013, it was reported that hyperglycemia induces mitochondrial fission by upregulating the expression of the dynamin-related protein 1 (Drp1) [83]. Drp1 is a cytosolic guanosine-5′-triphosphatase that triggers mitochondrial division by binding to fission 1 (Fis1) or to mitochondrial fission factor (Mff) in mitochondria. Increased mitochondrial fission contributes to DM-induced endothelial dysfunction. These studies suggest that suppression of mitochondrial fission can effectively prevent DM-induced atherosclerosis and its related cardiovascular complications [84].

### 1.8. Oxidative Stress in the Management of Type 2 Diabetes Mellitus

Metformin is a synthetic dimethyl biguanide very useful as a therapy for patients with type 2 DM. In addition to reducing blood glucose, metformin reduces cardiovascular complications in patients with DM, prevents the progression of the thickness of the intima media of the common carotid, and reduces the incidence of myocardial infarction in patients with type 2 DM [85, 86]. The beneficial cardiovascular effects of metformin appear to be independent of its antihyperglycemic effect because other conventional treatments such as insulin and sulfonylureas exhibit less beneficial cardiovascular effects. Increasing evidence has shown that metformin inhibits mitochondrial fragmentation (fission) in DM by activating AMPK resulting in preventing endothelial damage by activating processes such as apoptosis and inflammation [84]. In 2017, it was reported that metformin reduced Drp1 expression and Drp1-mediated mitochondrial fission in AMPK-dependent diabetic endothelial cells. Suppressing mitochondrial fission inhibits endothelial OS, improves endothelial function, and reduces atherosclerotic lesions [87]. Some studies show that metformin treatment can reduce MDA levels, increase GSH levels, and decrease inflammatory *status* [88, 89]. Metformin can decrease the production of ROS AMPK induced by decreasing ATP synthesis and NADPH oxidase activity [90].

### 1.9. Adjuvant Antioxidants in Diabetes Mellitus

In relation to the antioxidant state in DM, Lortz and Tiedge reported that overexpression of the enzyme SOD and Cat could protect the pancreatic islets from ROS and maintain insulin production. Similarly, GPx enzyme overexpression has been shown to protect INS-1 cells from ROS and attack by RNS [91]. Large-scale studies have shown that intensive early glucose control reduces the risk of micro- and macrovascular complications of DM [92]. Vitamin C, vitamin E, and *β*-carotenes have traditionally been considered as ideal supplements against OS and its complications in DM [80]. Milman et al. reported that vitamin E reduces cardiovascular events after 1.5 years of supplementation [93]. Blum et al. suggested that vitamin E supplementation in DM patients can prevent myocardial infarction, stroke, and cardiovascular death [94]. Akbar et al. performed a meta-analysis of 14 studies where they found that supplementation with antioxidants does not affect plasma glucose or insulin levels. However, the HbA1c level is significantly reduced by supplementation with antioxidants, apparently due to having a protective effect on DM complications [95].

Melatonin is an active indoleamine (derived from tryptophan) component with antioxidant properties secreted mainly by pinealocytes [96, 97]. The main function of melatonin is the regulation of the sleep cycle. Melatonin is also involved in homeostasis and energy metabolism [98]. Melatonin can activate brown adipose tissue, increase energy expenditure, and have anti-inflammatory, immunomodulatory, and antioxidant properties [99]. Melatonin also increases the expression of antioxidant enzymes (SOD, Cat, and GPx) and eliminate free radicals. Melatonin is indicated alone or in combination with other therapies for 1-3 weeks, where it can produce clinical improvement in patients with type 2 DM [100].

### 1.10. Oxidative Stress in Rheumatoid Arthritis

Increased OS has been found in mono- and polyarthritic rats [101]. Clinical evidence indicates that patients with rheumatoid arthritis have increased LPO, protein oxidation, and oxidative DNA damage [102]. Furthermore, ROS are positively associated with the severity of rheumatoid arthritis [103, 104]. Inflammation is the main pathophysiological mechanism of rheumatoid arthritis. Innate immune cells, such as neutrophils and macrophages, produce ROS, such as O_2_^·-^ and H_2_O_2_ [105]. Increasing evidence supports the link between the processes of redox reactions that produce OS and the pathophysiology of inflammation [106, 107]. Nuclear factor *κ*B (NF-*κ*B) is the transcription factor responsible for regulating different immune and inflammatory processes [108]. ROS can modify NF-*κ*B signaling in the cytoplasm and nucleus [109]. Nuclear translocation of NF-*κ*B can be induced by H_2_O_2_ and can be inhibited by overexpression of the SOD2 enzyme [110, 111]. Other transcription factors involved in cell differentiation, vascularization, and proliferation activator protein 1 (AP-1), inducible hypoxia factor (HIF-1), and gamma-activated peroxisome proliferator receptor (PPAR*γ*) are also induced by ROS [112–114]. ROS participate in the signaling of inflammation agonists. Mitochondrial ROS induce the production of proinflammatory cytokines, IL-1B, IL-6, and TNF-*α* [115]. The inflammation process also produces OS because polymorphonuclear neutrophils produce ROS through the NADPH oxidase enzyme pathway [116]. Furthermore, the ROS produced by the inflammatory cells condition a positive feedback of the inflammation [117].

### 1.11. Oxidative Stress in the Treatment for Rheumatoid Arthritis

Methotrexate is a folic acid antagonist originally used as a treatment for malignant diseases. Currently, methotrexate is one of the leading medications for the treatment of rheumatoid arthritis [118]. Methotrexate has immunosuppressive effects with mechanisms of action related to the generation of ROS. The increase in ROS by methotrexate is important for the cytotoxicity of T cells [119]. Methotrexate decreases enzyme levels of SOD, Cat, and total antioxidant activity and promotes apoptosis by increasing caspase-3 levels [120]. Inhibition of cellular NADPH has been suggested as one of the mechanisms of OS generation by methotrexate [121]. During the pentose cycle pathway, glutathione reductase uses NADPH as a reducing agent for cellular GSH (primary antioxidant). Decreased cellular GSH by methotrexate leads to reduced systemic antioxidant defense [122]. In addition, methotrexate generates mitochondrial dysfunction causing decreased activity of mitochondrial dehydrogenases, mitochondrial membrane potential, GSH, ATP concentrations, and increased LPO [123]. Methotrexate modifies the inflammatory response of different cells and cytokines with proinflammatory properties [124]. However, despite experimental evidence of methotrexate-induced OS, there is clinical evidence to suggest that methotrexate may have antioxidant activity. Some authors have shown that the management of rheumatic disease with methotrexate combined with glycosides reduces the levels of inflammation and OS [125]. Decreased LPO and increased GSH were observed in a study of female patients with rheumatoid arthritis in patients treated with methotrexate compared to patients without methotrexate [126].

### 1.12. Adjuvant Antioxidants in Rheumatoid Arthritis

Melatonin has been used as a protector from hepatorenal oxidative damage caused by methotrexate. Experimental studies have shown that the administration of melatonin reverses the increase in MDA, the activity of myeloperoxidase, and the decrease in GSH caused by methotrexate in the liver and kidney [127].

*α*-Lipoic acid has been used as a protective agent against methotrexate-induced liver OS. *α*-Lipoic acid is a coenzyme of pyruvate dehydrogenase naturally located in the mitochondria and used as a supplement for its antioxidant properties [128]. The administration of *α*-lipoic acid in mice showed decreased levels of LPO, protein carbonylation, and HO^**·**^ mitochondrial caused by methotrexate. In addition, *α*-lipoic acid restores antioxidant levels [129].

N-Acetylcysteine has also been shown to reverse the effects of methotrexate in decreasing GSH, SOD, and Cat and increasing MDA in liver samples [130]. In experimental models of rheumatoid arthritis, the endogenous antioxidant carnosine has been evaluated. Carnosine is a dipeptide with properties in the regulation of homeostasis, including protection against ROS, located mainly in the skeleton, cardiac muscle, liver, and central nervous system [131]. The combination of carnosine and methotrexate reduces the levels of LPO and C-reactive protein in plasma compared to methotrexate alone [36]. Combined therapy with methotrexate and vitamins A, C, and E has been shown to have better benefits in decreasing disease markers [132].

### 1.13. Oxidative Stress in Neurodegenerative Diseases

OS is associated with neurodegenerative diseases like Parkinson's disease [133], Alzheimer's disease [134], multiple sclerosis [135], and depression [136]. The main link between OS and neurodegenerative diseases is aging. OS accumulated during aging produces oxidative damage and gradual mitochondrial dysfunction [137]. Animal models with Alzheimer's disease show reduced activity of mitochondrial complex IV in the hippocampus [138]. Increased OS, in addition to causing direct mitochondrial oxidative damage, also produce neurotoxic subproducts. ROS favor the production of *β*-amyloid, a toxic peptide that participates in the neurodegenerative progression of Alzheimer's disease [139]. In addition, *β*-amyloid increases OS by activating H_2_O_2_ production in neocortical neurons [140]. Dysregulated activation of NADPH from microglia cells is also associated with neurodegenerative progress of dopaminergic neurons in Parkinson's disease models [141, 142]. The inflammatory and neurodegenerative activity associated with multiple sclerosis and depression is also linked to OS. In multiple sclerosis, an increase in the marker of oxidative damage to DNA (8-OHdG) and carbolinated proteins is found together with a decrease in the GPx enzyme [143]. On the other hand, high levels of MDA, decreased ascorbic acid, and SOD enzyme have been found in patients with unipolar depression [144].

### 1.14. Oxidative Stress in the Treatment of Neurodegenerative Diseases

Memantine is a glutamate N-methyl-D-aspartate receptor (NMDA) subtype antagonist used to decrease the neurodegenerative progression of dementia in Alzheimer's disease [145]. Memantine decreases the neurotoxicity of overactivation of glutamine receptors in the central nervous system [146]. Experimental memory deficit models demonstrate that memantine decreases protein oxidation in the hippocampus and cerebral cortex and reverses recognition memory deficit [147]. In addition, protective properties from oxidative damage have also been attributed to DNA primarily from the brain [148]. Memantine decreases levels of advanced protein oxidation products (AOPP) and advanced glycation end products (AGEs) in patients with prediabetes and cognitive impairment [149]. In addition, memantine can decrease nitrosative stress and increase antioxidant protection of nonprotein thiols in the cerebrospinal fluid [150].

Levodopa is a precursor to dopamine and is considered very effective for the symptomatic treatment of patients with Parkinson's disease [151]. Levodopa is often used in conjunction with carbidopa, a peripheral decarboxylase inhibitor, to increase the availability of levodopa by up to four times [152]. The activity of levodopa on the generation of OS has different postulates. On the other hand, *in vitro* evidence indicates that levodopa has neurotoxic properties induced by the generation of ROS [153]. Excess dopamine outside the synaptic vesicle caused by treatment with levodopa favors metabolism via monoamine oxidase or autooxidation, leading to the production of ROS. Spontaneous autooxidation of dopamine can produce O_2_ and reactive quinones [154]. However, models in lymphocyte cells have shown antioxidant effects of carbidopa/levodopa and protective properties against oxidative damage to DNA [155]. Use of the carbidopa/levodopa combination with other disease-related medications, such as monoamine oxidase inhibitors, has been shown to decrease the enzymatic metabolism of dopamine and levodopa by decreasing the generation of ROS [156]. This evidence suggests that the pro- or antioxidant characteristics of levodopa management are linked to fluctuations in dopamine metabolism that occur with treatment [157].

### 1.15. Adjuvant Antioxidants in Neurodegenerative Diseases

Some natural antioxidants have been used to enhance the antioxidant effects of pharmacology therapy. An experimental study reveals that the administration of ascorbic acid or rose oil can help to decrease the levels of oxidative damage to lipids or proteins induced by levodopa [158]. Studies show that the administration of vitamin E decreases the toxic effects of *β*-amyloid and improves cognitive development, decreases neuronal damage, and slows the progression of Alzheimer's disease [159, 160]. Green tea epigallocatechin gallate esters have inhibitory properties of amyloidosis and *β*-amyloid production both *in vitro* and *in vivo* [161]. Melatonin is another natural component that has been shown to have neuroprotective effects. In Parkinson's disease models, melatonin contributes to decreased dopamine production and decreases the LPOs and nitrites in the cytosol [162]. Melatonin has also been observed in clinical studies to improve sleep disorder in patients with Parkinson's disease, but not to improve motor symptoms [163, 164].

### 1.16. Oxidative Stress in Cancer

ROS have the ability to damage DNA and promote the development of carcinogenesis [165]. OH^·^ is the main ROS that attacks the mitochondrial and nuclear DNA strands producing different hydrolyzed base products such as 8-OHdG and 8-oxodG [166]. Cells can repair DNA damage by different enzyme mechanisms [167]. However, when DNA damage cannot be repaired, mutations related to base modification or deletion occur, leading to carcinogenesis [168]. The risk of poor DNA repair increases with the number of oxidative lesions that occur in DNA. Aging contributes to the accumulation of oxidative damage and decreased DNA repair [169]. Consequences of oxidative DNA damage include chromosomal abnormalities, blocking of DNA replication, and cytotoxicity [170, 171]. While oxidative damage to DNA is primarily caused by a direct free radical attack on DNA, free radical reaction with other cellular components may also contribute to mutagenicity [172]. LPO have carcinogenic capabilities [173]. MDA can react with guanine bases and form adducts [174]. All the mechanisms for the development of carcinogenesis caused by OS are still unknown. New mechanisms point to OS ability to alter the expression of genes and proteins involved in signaling cell growth and proliferation [175].

### 1.17. Oxidative Stress and Antineoplastic Drugs

Antineoplastic drugs have shown increased production of OS during the application of chemotherapy in cancer patients. Antineoplastic drugs promote the elevation of LPO and reduction of vitamins E and C and *β*-carotene [176].

*Doxorubicin* is a broad-spectrum anthracycline widely used in solid tumors [177]. Its mechanism of action is not completely known, but it consists of the inhibition of DNA and RNA synthesis, interfering with the activity of the enzyme topoisomerase II and the generation of ROS [178]. Doxorubicin has a quinone chemical structure that acts as an electron acceptor, producing a semiquinone radical that reacts with oxygen to form O_2_^·-^ and H_2_O_2_ [179]. The release of these free radicals increases OS causing DNA damage and cell death [180]. Despite the strong antineoplastic effects of doxorubicin, its use is limited due to its cardiotoxic capacity [181]. The main cardiotoxicity mechanisms of doxorubicin are OS and mitochondrial dysfunction [182]. Experimental evidence shows that treatment with doxorubicin increases OS in cardiac myocytes, causing accumulation of irreversible cardiotoxicity [183]. Doxorubicin increases the production of O_2_^·-^ and NO^·^ by joining the eNOS reductase domain [184]. eNOS is the major NOS isomorphism involved in the development of left ventricular dysfunction induced by doxorubicin [185]. Some studies have proposed using antioxidants to decrease the cardiotoxicity of doxorubicin. The cardioprotective effects of coenzyme Q10 have been evaluated in pediatric patients on anthracycline therapy. Patients receiving coenzyme Q10 were reported to show benefits in cardiac function [186].

Cisplatin is one of the main representatives of the drugs in the group of coordination complexes with platinum used for several decades to treat different types of cancer [187]. Cisplatin anticancer activity consists of the ability of platinum to form covalent adducts with nuclear DNA. These cisplatin-DNA junctions form crosslinks between the outer and inner strands causing the strands of nuclear DNA to break. DNA damage ends up, causing cellular apoptosis [188]. Like other cancer drugs, the use of cisplatin is also limited by its side effects. One of the main toxic effects is nephrotoxicity [189]. OS represents an important mechanism of tissue damage from the use of cisplatin. Cisplatin-induced nephrotoxicity is associated with mitochondrial damage represented by decreased GSH, oxidative damage of lipids and mitochondrial proteins, and increased apoptosis [190]. MDA has been proposed as a predictor of the development of cisplatin-induced kidney failure [191]. Increased liver concentrations of LPO products are also related to cisplatin-induced hepatotoxicity [192]. High doses of cisplatin cause mitochondrial OS and damage to liver energy metabolism [193].

### 1.18. Adjuvant Antioxidants in Cancer

Coenzyme Q10 (ubiquinone) is not FDA approved to treat any medical condition. However, it is widely available over the counter as a dietary supplement. Chronic diseases like cancer, neurodegenerative disease, fibromyalgia, DM, mitochondrial diseases, muscle diseases, and heart failure are associated with decreased circulating levels of coenzyme Q10 [194]. Coenzyme Q10 is a fat-soluble vitamin-like molecule that occurs naturally in every cell membrane in our bodies. It is a normal part of our diet, but it is also synthesized endogenously. It is essential for the proper transfer of electrons within the mitochondrial respiratory chain and the production of adenosine triphosphate (ATP) [195]. Coenzyme Q10 has the ability to increase the production of key antioxidants such as SOD. The coenzyme Q10 reduces LPO levels by reducing prooxidant compounds and is capable of improving blood flow and protecting blood vessels through the preservation of NO^·^ [196]. Coenzyme Q10 is safe as a dietary supplement. Toxicity is unlikely, even up to a daily intake of 1,200 mg/day. The typically studied doses have been from 100 to 200 mg/day [197].

Resveratrol (3,5,4′-trihydroxy-trans-stilbene) is a polyphenolic phytoalexin present in a variety of plant species such as peanuts, grapes, berries, and red wine [198]. Preclinical studies have shown that resveratrol has protective effects in various disease models, such as DM and cancer [199]. Resveratrol *in vitro* systems have been shown to directly remove a variety of oxidants, including the OH^**·**^ radical, O_2_^·-^, H_2_O_2,_ and ONOO^−^. In a cell-free system using the Fenton reaction as the OH^·^ source, resveratrol (at concentrations ≥ 300 *μ*M) has been shown to act as a scavenger rather than an inhibitor of the Fenton reaction. The calculated reaction rate of resveratrol of OH^·^ (9.45 × 108 M^−1^·s^−1^) is significantly less than that of well-established antioxidants, including ascorbate (1.2 × 1010 M^−1^·s^−1^), glutamate (GSH) (1.5 × 1010 M^−1^ S^−1^), and cysteine (1.3 × 1010 M^−1^·^−1^). The property which has been proposed to remove OH^·^ of resveratrol is due to its phenolic groups [200]. Resveratrol (at concentrations ≥ 100 *μ*M) has been shown to remove the radical O_2_^·-^ directly in a nonenzymatic, cell-free system (potassium O_2_^·-^ system) [201]. Resveratrol (10 *μ*M) increases mitochondrial mass and mitochondrial DNA and regulates constituents of the electron transport chain and mitochondrial biogenesis factors in cultured coronary artery endothelial cells in humans [202]. Very high doses of resveratrol (up to 3000 mg) have been used in some clinical trials. However, low doses (5 mg in humans or 0.07 mg·kg^−1^ in mice) have been shown to have even superior chemopreventive efficacy against cancer at high doses (1000 mg in humans or 14 mg·kg^−1^ in mice) [203].

### 1.19. Oxidative Stress in Antiretroviral Therapy

The introduction of highly active antiretroviral therapy (HAART) has reduced the morbidity and deaths associated with human immunodeficiency virus infections (HIV) [204]. Drugs classified as nucleoside or nucleotide reverse transcriptase inhibitors (NRTI or NtRTI), nonnucleoside reverse transcriptase inhibitors (NNRTI), protease inhibitors (IP), integrase inhibitors, and fusion inhibitors/entry are traditionally used in the treatment of HIV infections. Current HAART administration guidelines recommend the combination of two NRTIs, an NNRTI, or a protease/integrase inhibitor, depending on the patient's efficacy and tolerability. NRTIs (abacavir, didanosine, lamivudine, stavudine, zidovudine, and emtricitabine) act as false substrates that sabotage the lengthening of the viral cDNA chain, inhibiting viral reverse transcriptase activity by limiting viral replication [205]. NRTIs are associated with hepatotoxicity, such as steatosis, steatohepatitis, disorders of lipid regulation, enlarged liver, and abnormal liver functions [206], although the specific mechanisms through which complications of NRTIs occur have not yet been clearly defined. NRTIs have been shown to inhibit *γ*-DNA polymerase, leading to mitochondrial DNA depletion and mitochondrial toxicity, leading to impaired oxidative phosphorylation and oxidative damage to cellular machinery, along with delayed cell cycle progression resulting in apoptotic cell death [207]. These effects have been attributed to the binding of NRTI-triphosphate (the active metabolite of most NRTIs after intracellular phosphorylation) to replicating mitochondrial DNA that causes the termination of viral chain elongation [208]. The marked increase in MDA, end products of LPO, and protein carbonyls has been associated with the administration of NRTI, together with a decrease in the activity of enzymatic antioxidant proteins as a consequence of the disorder of the oxidative phosphorylation process [209]. Known metabolic complications of NRTI administration include lipodystrophy, dyslipidemia, hepatotoxicity, hepatomegaly, metabolic syndrome, increased lactic acid, and cardiomyopathy [210]. Oxidative cell damage caused by mitochondrial toxicity is one of many scientific mechanisms that underline the development of complications from NRTI [211].

On the other hand, active HIV infection in the central nervous system is undoubtedly a factor that contributes to the development of cognitive deficit [212]. Stopping viral replication in brain tissue and the rest of the body is essential for prevention. However, the potential of antiretroviral treatments to contribute to this degenerative condition has not been fully explored in clinical studies or in experimental models. NRTI are essential drugs in most combination antiretroviral therapy (cART) regimens. The most common side effects of these medications that limit clinical use are myopathy, lactic acidosis, and peripheral neuropathy. All of which are closely related to mitochondrial toxicity. The implementation of cART has dramatically increased the survival rate of people infected with HIV and has almost completely prevented severe dementia associated with the virus [213, 214]. The putative molecular mechanism that governs NRTI-mediated mitochondrial toxicity is the specific inhibition of mitochondrial polymerase *γ* (pol *γ*) [215]. Because pol *γ* is the primary DNA polymerase in mitochondria, inhibition of pol *γ* is expected to lead to reductions in mtDNA synthesis and subsequently to reductions in the supply of critical protein subunits of respiratory complexes of the electron transport chain. Deficiencies in these proteins should cause decreased ATP production and accumulation of orphan respiratory complex subunits encoded by nuclear DNA. Despite the high correlation between pol *γ* inhibition *in vitro* and the severity of clinical side effects, studies in cell culture have shown that mitochondrial dysfunction can occur in cardiac myocytes or hepatocytes independent of mtDNA depletion [216]. When NRTI interfere with the action of mitochondrial DNA polymerase, mitochondrial replication is inhibited. This gradually reduces mitochondrial function in various tissues that is evident primarily in metabolically active organs such as the heart and liver, resulting in cardiotoxicity and heap toxicity [208].

### 1.20. Natural Antioxidants in HIV

Common HIV antioxidants such as vitamins C and E, uridine, and carnitine have been investigated to prevent or reverse complications from NRTI management with minimal success [217]. Therefore, further research is needed for alternative antioxidants that may be more effective in controlling complications of NRTI. Dietary and nutritional therapies are viable options that have not been vigorously applied. The beneficial effects of some currently available antioxidants have been used in animal models, but large-scale validated clinical trials are still lacking [218]. Plant-derived flavonoids such as naringin (4′,5,7-trihydroxyflavone 7-rhamnoglycoside) are commonly found in citrus. Naringin has been recommended as beneficial to reduce the risk of DM and CVD in predisposed populations [219]. The antioxidant capacity of naringin has been demonstrated through its action in the elimination of free radicals, antiapoptosis, antihyperglycemic, antimutagenic, anticancer, anti-inflammatory, and cholesterol-lowering agents [220]. HIV causes symptoms that are similar to those of NRTI-induced metabolic complications. In 2015, the authors reported an experimental study in mice where naringin reversed the metabolic complications associated with NRTI by improving OS and apoptosis. This evidence implies that naringin supplementation could mitigate lipodystrophy and dyslipidemia associated with NRTI therapy [221]. Naringin is a cheap and readily available dietary flavonoid in most citrus fruits with proven antioxidant and antiapoptotic properties that have shown favorable effects in animal models *in vitro*, *in vivo*, and *ex vivo*. The mechanism by which naringin improves metabolic complications possibly implies its antioxidant and/or antiapoptotic effects [222]. The mechanism of action is worth further investigation in patients treated with NRTI through well-conducted clinical studies, where naringin is administered at different doses.

## 2. Conclusions

OS is closely linked with the pathological mechanisms of different chronic diseases. The role of pharmacological therapy on OS depends both on the chemical characteristics of the active molecules and on the consequences of the mechanisms of action. Medicines such as CCB have a dihydropyridine ring that gives them antioxidant structural characteristics. On the other hand, other antihypertensive drugs show beneficial antioxidant activity as a result of regulating the antihypertensive mechanism to normal. Immunosuppressive and antiretroviral drugs are the treatments that cause the most oxidative damage in patients in the long term, and antioxidant management alternatives are very limited in experimentation or with insufficient results to treat these pathologies. The investigation of the oxidative mechanisms of these pathologies and of the conventional medicines used to treat them will allow a better understanding, monitoring, or selection of alternative antioxidant medicines according to the health condition of each patient to decrease oxidative damage.

GSH: glutathione; SOD: superoxide dismutase; MDA: malondialdehyde; AGEs: advanced glycation end products; NRTI: nucleoside reverse transcriptase inhibitors.

## Figures and Tables

**Table 1:** Antioxidant alternatives in the management of chronic diseases.

Antioxidant	Chronic disease	Results	Reference

N-Acetylcysteine	Atherosclerosis	Prevents the progression of atheroma in uremic mice	[44]
Paricalcitol (vitamin D)	Atherosclerosis	Enalapril and paricalcitol decrease MDA and increase GSH; affords greater protection against aortic inflammatory injury in mice	[45]
Naringin	HIV infection	Naringin reverses the metabolic complications associated with NRTI by improving OS and apoptosis in a rat model	[221]
Vitamins A, C, and E	Rheumatoid arthritis	Combined administration of vitamins A, B, and C with methotrexate for 10 weeks lowers the severity score in patients with rheumatoid arthritis	[133]
Ascorbic acid and essential oil rose	Parkinson's disease	Ascorbic acid or essential rose decreases MDA, AGEs, and carbonyl concentration of mice treated with levodopa	[159]
Vitamin E	Alzheimer's disease	Vitamin E delays the progression of disease in patients with Alzheimer's disease	[161]
	Type 2 diabetes mellitus	Vitamin E increases event-free survival in type 2 diabetes mellitus patients	[93]
Coenzyme Q10	Hypertension	Increase SOD levels and decrease MDA in hypertensive elderly subjects	[197]
	Lymphoblastic leukemia	Treatment with coenzyme Q10 provides a protective effect on cardiac function during treatment with anthracycline in patients with lymphoblastic leukemia	[187]
